# Plant spraying quality when used by drone-robots

**DOI:** 10.1038/s41598-026-40649-6

**Published:** 2026-02-26

**Authors:** Bogusława Berner, Jerzy Chojnacki, Leon Kukiełka, Jan Najser, Jan Kielar, Tomáš Najser, Leszek Sergiel

**Affiliations:** 1 Pomeranian Agricultural Advisory Centre (PAAC – PODR), Lubań, Poland; 2 ENET Centre, CEET, VSB-Technical University of Ostrava, Ostrava, Czech Republic; 3 Faculty of Mechanical Engineering and Energetics, Koszalin University of Technology, Koszalin, Poland; 4 Institute of Technology and Life Sciences – National Research Institute (ITP – PIB), Falenty, Poland

**Keywords:** UAVs, Environmental protection, Food and energy crops, Air flow, Spray deposition, Leaf area index (LAI), Ecology, Ecology, Engineering, Plant sciences

## Abstract

The study aimed to evaluate how airflow from a small drone’s rotor affects the accuracy and consistency of liquid coverage on individual plants or small plant groups during low-altitude spraying. The research focused on the future possibility of using drones - unmanned aerial vehicles (UAVs) as autonomous robots for plant protection. The effects of UAV propellers’ rotation on changes in droplet stream, produced from a single spray flat fan nozzle, as well as the influence of altitude, flight speed, and leaf area index (LAI), on the liquid coverage uniformity (CU) of application on plants were examined. The rotational speeds of the UAV propellers were set at 0.0, 5000, and 6400 rpm. Rapeseed and potato plants were selected for testing because they are common food crops and sources of raw materials for biofuel production. The UAV, on a laboratory test stand, was moved at two heights (H = 0.5 and 1.0 m) at two speeds (v = 0.54 and 1.0 m·s^-1^). The airflow from the UAV’s rotors narrowed the droplet stream produced by the nozzle by about 20% and increased the liquid volume in the center of the droplet stream, especially at a UAV height of H = 1.0 m. The leaf area index (LAI) for rapeseed was 0.877, and for potatoes it was 6.273. These LAI differences resulted in the liquid sprayed from the UAV settling more evenly on rapeseed plants than on potato plants. UAV flight altitude and leaf area index (LAI) appeared to be key factors affecting the amount of liquid applied to plants. Lower altitudes (H = 0.5 m) improved liquid application uniformity and enabled deeper penetration into dense foliage. High LAI values significantly hampered liquid penetration into lower plant levels and reduced the uniformity of liquid application.

## Introduction

In recent years, a new form of agricultural aviation has rapidly evolved involving the use of drones - unmanned aerial vehicles (UAVs) - in agricultural applications. Unmanned aerial vehicles are employed there by both specialized service companies and farmers. Their primary applications include monitoring crop health, assessing nutrient requirements, and detecting symptoms of biotic and abiotic stress^1–3^. Additionally, UAVs are increasingly employed for field treatments such as spraying plants against pests^4,5^, sowing seeds^6^, and spreading mineral fertilizers^7^.

Among UAV designs used in agriculture, multicopters dominate because of their ability to hover and independently adjust altitude and flight speed, providing high operational flexibility^8,9^. One key advantage of using UAVs for spraying is the reduction of plant damage and soil compaction, which are often unavoidable with ground-based equipment. UAVs can also perform treatments in areas that are difficult or inaccessible for traditional field sprayers, such as wetlands, mountainous regions, and steeply sloped areas where plants are cultivated. Multirotor (multicopter) unmanned aerial vehicles are also particularly useful for protecting tall crops, such as sugarcane and corn^10–13^.

To improve the economic efficiency of plant protection relative to sprayers, research is ongoing to optimize treatment parameters, such as UAV altitude and speed, enabling faster and more efficient applications^14^. For example, extending the altitude from 5 to 11 m above the crops during UAV spraying provides a broader coverage of the crop with liquid^15–18^. Concurrently, manufacturers of commercial UAVs are introducing models with increased tank capacities, reaching up to 100 l, and capable of flying at speeds up to 20 m·s^− 1^, e.g., DJI AGRAS T100^19^. However, the pursuit of liquid coverage of the maximum field area in a short time often leads to the use of fine droplets and higher concentrations of agrochemicals, which can compromise uniformity of plant coverage, increase droplet drift, and thereby increase elevated environmental risks^20^.

A distinctive feature of multicopter UAVs, compared with conventional sprayers, is the airflow generated and shaped by rotors configuration and number. This downward air stream can significantly affect liquid deposition quality and, when properly managed, can also decrease droplet drift^21–23^.

To optimize airflow for improved liquid application during spraying, computational fluid dynamics (CFD) methods are widely employed. These simulations analyze the relationship between rotor-generated air stream and spray droplet trajectories, providing insights into their combined impact on deposition quality^24–31^. CFD results are typically validated by comparing them with actual measurements of airflow and liquid deposition on plants^27–29^. These studies have identified phenomena such as droplet and flowing air mass reflection off plant surfaces, as well as the formation of horseshoe vortices, which induce turbulence in the air. These turbulences can secondarily lift up falling droplets, increasing their ability to drift over long distances, and can raise concerns about environmental contamination and human health risks^25^. One of the most effective ways to prevent these phenomena is to spray crops with UAVs at the lowest feasible altitude and appropriately chosen speeds^9,32–34^.

In addition to field studies and numerical simulations, experiments are also conducted under controlled laboratory conditions^35–37^. These tests allow for detailed observation and measurement of the droplet stream and airflow produced by the UAV, as well as factors affecting the drift of the sprayed liquid. They also provide an accurate assessment of the amount of liquid deposited on the plant surface. Their main advantage is the ability to eliminate the influence of atmospheric factors such as wind or temperature changes, resulting in more repeatable and precise results. Additionally, they allow the artificial creation of atmospheric conditions similar to those in the field in a stable and controlled manner^37,38^. The outcomes of laboratory tests are a valuable supplement to the knowledge gained from field experiments and numerical analyses.

Thanks to the ability to maneuver precisely over crops, UAVs are becoming a crucial tool in precision and sustainable agriculture. When combined with artificial intelligence algorithms, including neural networks, they can be seen as part of the emerging concept of smart agriculture^39–43^. A particularly important development is the integration of two functions in unmanned systems: (i) observing and assessing crop conditions concerning pest threats, and (ii) autonomously selecting areas for applying plant protection products. Solutions like these will establish UAVs as intelligent flying robots capable of targeted pest control^44^. However, the complexity of issues surrounding UAVs use in agriculture presents a technical and scientific challenge for maximising the efficiency and accuracy of treatments^45^.

Research and implementation projects for observation UAVs are increasingly using advanced technologies, such as lasers, multispectral and hyperspectral sensors, and AI-based image analysis systems^46^. These technologies not only allow the identification of pest-affected crop areas, but can also accurately pinpoint individual plants requiring intervention. UAVs equipped with such solutions could spray variable doses of chemicals, tailored to crop size and development, as well as specific agronomic and biological requirements.

### UAVs in precision farming

Autonomously operated UAV flights at low altitudes over crops, used for both precise monitoring and spraying, may become a common tool in modern agriculture in the future. This technique provides a foundation for developing accurate systems for spot plant inspection and pest control. Accurate identification and spraying of individual plants require determining the plant’s size and developmental stage, as well as the drone’s precise position relative to the plant. The identification of the UAV’s position relative to individual plants can be aided by digital field maps, on which, according to GIS (Geographic Information System), individual plants are precisely marked. In this context, seeders and planters have already been developed that allow the geographical position of each sown seed, as a future plant, to be recorded^47,48^. UAVs equipped with RTK modules and advanced optical sensors can use such maps to perform precision crop inspections and spot-treat selected plants^45,49^.

To achieve accurate liquid deposition on plants, the UAV should fly as close as possible to the crop surface, and the stream of the applied agent should be directed straight at the plant being treated. A useful parameter for setting spray parameters is the Leaf Area Index (LAI), which helps to assess the size and condition of cultivated plants^50^. This index is already used to evaluate the effects of spraying with ground sprayers^51^. Similar efforts are underway to improve UAV-based spraying quality^52^.

Conducting a thorough inspection of plants and applying precise, selective spraying treatments requires slower flight speeds than typical high-efficiency UAV spraying. The duration UAVs can stay in the air is limited by their battery capacity; however, they can be recharged in the field with solar-powered docking stations, which enable them to automatically land and connect^53,54^.

The key challenge in using UAVs for precise pest identification and control is planning their flight routes. Before designing the route, the following factors must be considered: task type, operational area size, varying flight altitudes, obstacles, interactions among multiple vehicles in the same airspace, and dynamically changing environmental conditions during flight. The UAV’s energy requirements are also important, particularly the energy reserve required to return to the launch site or reach the nearest docking stations.

Typical UAV flight route planning involves setting the altitude and speed and marking points (waypoints) on the control station screen that the UAV should visit during the task. Obstacles visible on digital field maps that may block flights are also avoided. These are most often trees, poles, power lines, and buildings^55^.

For observation UAVs, camera specs are also necessary to plan the route^56^. When planning precision spraying in designated areas of the field, many key parameters must be taken into account, such as: constant or variable liquid flow rate from the nozzles and the number of nozzles used in spraying, the required dose of spray liquid on the surface of the sprayed plants or crop area, the shape of the sprayed liquid stream, the width of the sprayed swath, the speed of the UAV, the influence of wind, the accuracy of positioning in relation to the plants, and the minimum safety distance from fixed and moving obstacles^57^. To enable the UAV to spray only selected areas in the field and follow the shortest route, without returning to the same locations and with minimal energy and liquid consumption, methods of flight optimization are being sought. To this end, research efforts are underway to deploy advanced methods on the UAV, both offline and online, including mathematical procedures and neural networks^58^. Since the methods traditionally used in UAVs, which employ ultrasound to avoid obstacles, are insufficient, efforts are underway to enable unmanned aerial vehicles, especially those operating at low altitudes, to ‘see’ objects in fields. For object detection, the potential of neural network algorithms is also being investigated^59^. To this aim, deep learning methods, such as YOLOv3, which are widely available through open-source implementations. When integrated with satellite- or markers-based positioning systems, these methods enable the determination of a UAV’s position relative to surrounding objects^60,61^. Additionally, research is underway to apply perception-based approaches to acquire information about objects in environment. These approaches are based on collecting data about objects in the environment using various sensors (cameras, radars, microphones, and temperature sensors) installed on UAVs, and then identifying and interpreting objects in a manner inspired by human or animal perception systems^62^. It is desirable to use “depth cameras” and neural networks for this purpose. The “depth cameras” not only transmit images but also provide distance data for individual recorded objects relative to the UAV in flight^63^. Using deep learning algorithms, the UAV learns to autonomously avoid obstacles, including those that appear unexpectedly^64^. The equipment of the flying UAV should not only provide it with information about obstacles in its flight path, but also enable it to make decisions and perform maneuvers in such a short time that it can avoid contact with them^18,65^.

In all UAV observation missions over crops, even when it is only collecting data for the Internet of Things (IoT) from sensors deployed in the field, it is not only important to visit as many points as possible, but also the economical use of memory capacity and battery capacity^66^. Therefore, optimising the planned route to minimise travel time and maximise the amount of information obtained is particularly important. This issue affects not only observation missions themselves but also their integration with autonomous spraying, such as when managing detected weeds or pests, and it also affects spraying missions exclusively^60,67^.

Another challenge in route planning is managing multiple UAVs in the field, avoiding collisions, and running trajectory-planning algorithms on limited UAV computing power in real time^68,69^. It should be noted that the use of UAVs as robots, equipped with artificial intelligence to control their trajectories during fieldwork, raises safety concerns for them and increases their susceptibility to external attacks and disruptions^70,71^. Control system failures that lead to unpredictable events may also happen. It is essential to protect unmanned aerial systems against such risks^72^.

In a precision farming system, using flying robots, the quality of spraying selected plants relies on the high accuracy and uniformity of liquid application. The primary advantage of precision spraying, for individual plants at risk of pathogen attack or already attacked should be to deliver treatment only to areas that need it, thereby improving food quality and its safety by reducing unnecessary pesticide use where it is not needed. This is especially important when plants are meant solely for consumption. In the case of UAVs used to protect crops for energy production,, the more important factor in the precision of spraying is the economic benefit of reducing unnecessary chemical use.

Performing treatments at the lowest possible UAV altitude above the crops not only reduces the risk of environmental contamination by minimizing droplet drift, but also allows the UAV’s airflow to enhance the quality of liquid application to the plants. In that case, precise spraying of individual plants or small groups can be achieved with a nozzle aimed directly at them, similar to manual spraying. When UAVs fly at low altitudes over plants, there is also a risk that sudden gusts of wind may cause variations in the amount of liquid applied to the plants and spray drift or UAV position change. To prevent such issues, intelligent systems to improve their positioning and spray uniformity are being tested^73^.

Previous studies on optimizing aerial spraying parameters indicate that factors such as plant species and morphology, pest type, UAV weight and design, flight altitude and speed, and the type size of spray nozzles used influence treatment effectiveness and the quality of liquid deposition on crops^10,15,74–77^.

Many nozzle designs currently installed in unmanned aerial vehicles have been in use for years in field sprayers and manned aircraft. One of the simplest and most common solutions is to use pressure nozzles, especially flat fan nozzles^76^, and hollow-cone or full-cone nozzles^33^. These nozzles are usually mounted directly beneath propeller rotors or on tubular beams suspended below them^78^. This solution causes airflow from the rotating propellers to push droplets downward, accelerating their movement and improving the penetration of the sprayed liquid into the plant canopy.

The size and shape of the droplets produced, as well as the size and shape of the sprayed liquid stream from pressure nozzles, depend on the size and shape of the outlet opening, the internal design of the nozzles, and, especially, the pressure of the liquid generated by the pump mounted on the UAV.

The size of the droplets produced, as well as the shape and size of the sprayed liquid stream from pressure nozzles, depends on the size and shape of the outlet opening, the internal design of the nozzles, and, especially, the pressure of the liquid generated by the pump mounted on the UAV. The design of pressure flat-fan nozzles produces a thin, fan-shaped liquid stream, whose width depends on the spray angle and the UAV’s height above the plants^79,80^.

Creating fine and very fine droplets from pressure nozzles can increase their drift caused by air currents^76,78^. In studies on reducing the drift of liquids sprayed using UAVs, Air Induction (AI) pressure nozzles have proven to be highly effective compared to typical pressure nozzles^23,79–81^. Due to their design, these nozzles additionally suck in air before forming droplets. This phenomenon produces liquid droplets filled with air bubbles. They break down into smaller droplets only upon reaching the plants, and are less prone to drifting than droplets from conventional pressure nozzles^80,82^. Air Induction nozzles can also be manufactured in flat-fan versions with single- and double-stream configurations^4^. The use of flat-fan AI nozzles in UAVs can increase the liquid coverage of plants by more than twice what is achieved with conventional flat-fan nozzles of the same size^83^.

In addition to pressure nozzles, centrifugal atomisers are increasingly used in UAVs. These nozzles spray liquid through discs that spin at high speeds. Their main advantage is their ability to control droplet size independently of liquid flow rate, in following the Control Drop Application (CDA) method^13,81,84^. The centrifugal atomisers also allow high liquid flow rates. This rotary spraying system can vary droplet size between 40 and 1000 μm by adjusting the rotation speed of the spray discs to the liquid flow rate^85,86^, but it also changes the width of the droplet stream.

Nozzles that rotate in UAVs generally carry a higher risk of droplet drift compared to pressure nozzles^87^. However, studies in^88^ indicate that droplet drift from rotary sprayers may be comparable to that from air induction nozzles. Efforts are also progressing to enhance droplet deposition onto plants by electrifying rotary sprayers^89^. Centrifugal atomisers in UAVs generally pose a higher risk of droplet drift compared to pressure nozzles^87^. However, studies in^88^ suggest that droplet drift from centrifugal atomisers may be comparable to that from air induction nozzles. Efforts are also underway to improve the deposition on plants by electrostatically charging droplets produced by centrifugal nozzles^89^.

In precision spraying of individual plants or small groups of plants using a UAV, selecting an appropriate spray nozzle can be critical to treatment effectiveness. Spraying at a height of 0.5 to 1.0 m above the plants may require the use of a single nozzle. The choice of nozzle will depend on whether the spray should cover only the plant being sprayed, e.g., weeds, or the plant on which the parasite is being destroyed, or whether preventive spraying of neighboring plants should be carried out at the same time. When using pressure nozzles, the droplet stream width is determined by the spray angle, which depends on the nozzle design. The spray angle of liquid released from the UAV can also be influenced by airflow generated by the propellers^35^. The size of the droplet stream from the centrifugal atomizer is more difficult to control, as its operating width depends on the liquid flow rate, the atomization process, and the disc’s rotational speed.

### Research objectives

The primary objective of these studies was to examine the factors affecting the accuracy and quality of crop spraying using an unmanned aerial vehicle, performed at low altitudes above the plants, similar to the typical spray boom height used with ground-based field sprayers. This research focused on the feasibility of spraying with a single nozzle mounted below one of the UAV’s rotors.

The specific aim was to evaluate how the airflow generated by the small UAV’s rotors influences the volume of liquid that settles at plant levels and to analyze the consistency of liquid coverage across all plant levels. Additionally, the study aimed to evaluate the effect of Leaf Area Index (LAI), a physical and mathematical indicator characterizing the sprayed plants, on the uniformity of liquid application to plant surfaces during aerial spraying.

Another important objective, useful for explaining phenomena that may occur when using UAVs, was to measure air velocity in the airflow beneath the drone’s rotors and to assess how this airflow affects the distribution of liquid within the droplet stream produced by an individual nozzle.

## Materials and methods

### Test stand

**Fig. 1:**
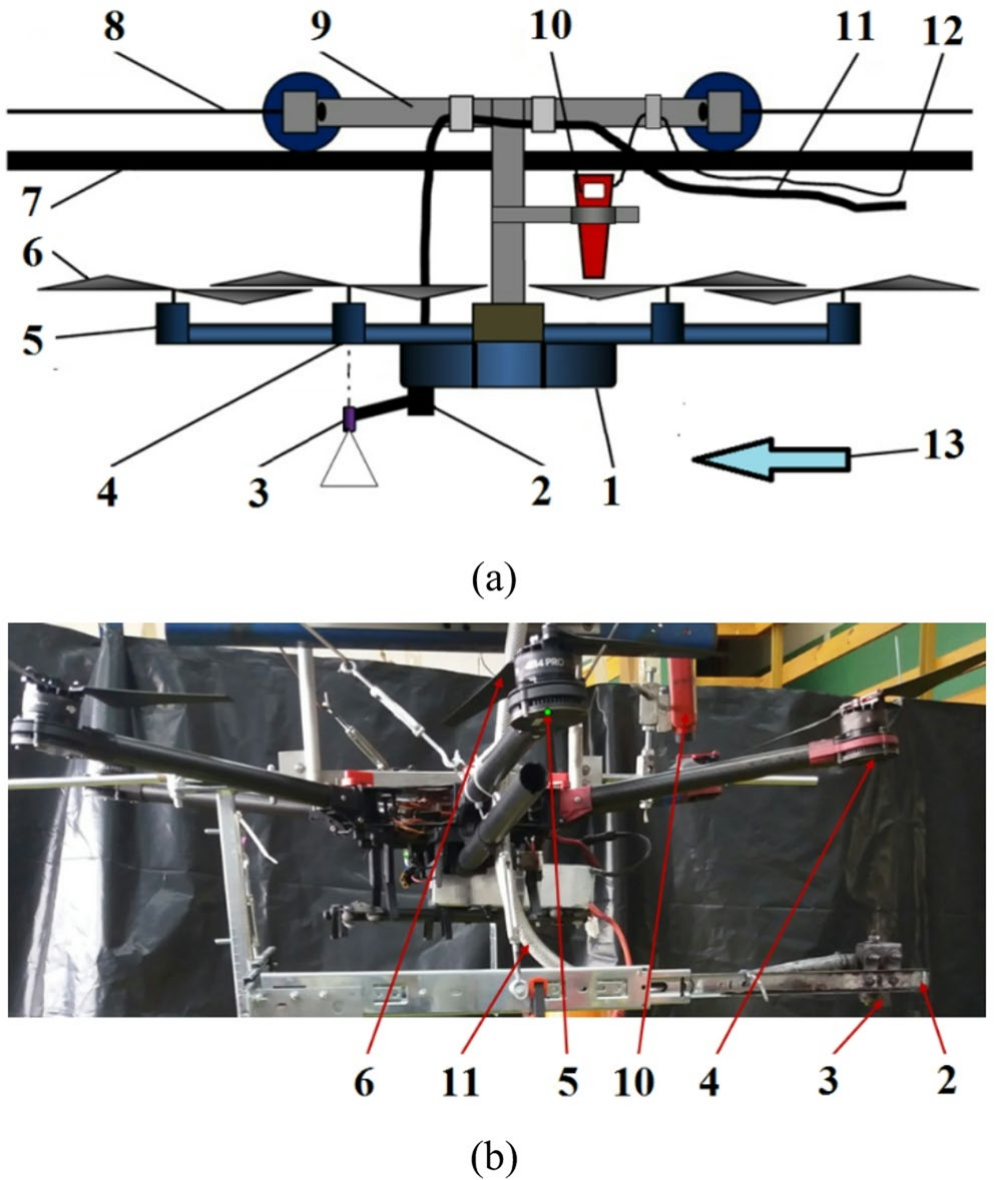
Test stand for moving the UAV over plants: **a** diagram of the test stand, **b** front view of the UAV: 1 – frame base, 2 – adjustable bracket, 3 – spray nozzle, 4 – first side rotor, 5 - front rotor of the drone, 6 – propellers, 7 – track, 8 – tow rope, 9 – trolley, 10 – tachometer, 11 – liquid pipe, 12 – USB cable, 13 – direction of drone movement.

To eliminate the influence of weather conditions on the test results and ensure the repeatability of unmanned aerial vehicle flight parameters, such as route, altitude, and flight speed, the experiments were conducted under controlled laboratory conditions. For this purpose, a special test stand was prepared to move the UAV over the plants, the diagram of which is shown in Fig. 1.

As a multi-rotor UAV, a DJI S-900 ─ hexacopter was used for the tests. Their frame frame consisted of six arms attached symmetrically to the base (1), with rotors mounted at their ends. These were brushless electric motors with a maximum power of 500 W, which had rotating propellers (6) measuring 15 × 5.2”. The UAV was mounted on a trolley (9) moved by means of a tow rope (8), wound with the help of a rotating wheel mounted on the electric motor shaft. The trolley’s speed was controlled by adjusting the frequency of the electric current supplied to the motor. A spray nozzle (3) was mounted on an adjustable bracket (2) under the first side rotor (4) in the direction of the drone’s movement (Fig. 1(a) and (b)). The distance between the nozzle outlet and the lower surface of the propellers was 0.15 m. The axis of its symmetry coincided with the axis of symmetry of the rotor with propellers under which it was located.

When selecting the minimum altitude for the UAV above the plants, the average spray nozzle height of 0.5 m used with a ground-based field sprayer was taken into account, along with the likelihood of reducing droplet drift at this height. The UAV height above the plants was measured as the distance from the spray nozzle to the plant tops. Two heights were used in the tests: H = 0.5 m and H = 1.0 m, and two travel speeds: v = 0.54 and v = 1.0 m·s^− 1^.

After analyzing test results from spray nozzles used in commercial and research UAVs for plant spraying, a single Lechler ST 110-02 flat-fan spray pressure nozzle was selected for further testing. These nozzles are commonly used in commercial unmanned aerial vehicles. The flat, produced shape of the droplet stream from these nozzles enables precise spraying of specific areas within the field or on individual plants. This type of pressure spray nozzle also allows for fine atomization of the liquid at low pressure. It was assumed that at low flight altitudes, airflow can significantly displace fine droplets deep into the canopy of the plants. When selecting a nozzle with a wide spray angle, the possibility of common preventive spraying of neighboring plants with a smaller liquid volume was also considered.

The liquid was supplied to the nozzle through a plastic pipe (11) connected to a small stationary sprayer. The liquid flow rate from the nozzle was determined experimentally at a pressure of *P* = 0.2 MPa, as used in all experiments, and amounted to V = 0.63 l·min^− 1^. The UAV’s propeller rotation speed was manually controlled from a control station and measured using an optical tachometer (10) connected to a computer via a USB cable (12).

To simulate the UAV’s flight, even when it is moving on a trolley, the propellers should operate at a rotational speed sufficient to generate thrust equal to the UAV’s weight. For this purpose, the rotors’ rotational speed was selected experimentally, taking into account the UAV’s assumed weight. The overloaded unmanned aerial vehicle was placed on an electronic scale. At the same time, its weight loss was measured with an electronic scale, and the propeller rotation speed was measured using an optical tachometer. Two UAV weights were assumed for the tests: one with an empty spray-liquid tank and the other with a full tank, because it was believed that filling the tank could significantly affect the quantity and quality of the liquid deposited on the plants. The weight of the drone with a full tank was set at m = 12.5 kg, which corresponded to a rotor speed of *n* = 6400 rpm, while the mass of the drone with an empty tank was set at m = 7.4 kg, which corresponded to *n* = 5000 rpm. These are parameters characteristic of the smallest drones used in plant protection (https://www.ttaviation.org/pro/tta-m4e-5 l-agriculture-drone/). It was decided that at spraying heights H < 1.0 m, the use of heavy UAVs is not recommended due to the risk of plant damage from the rotor air stream. To compare the obtained results and assess the impact of airflow on the quality of the applied liquid, the experiments also included spraying the plants with the rotors stationary, without airflow.

### Measurement of the air velocity in the airflow generated by the rotors

**Fig. 2:**
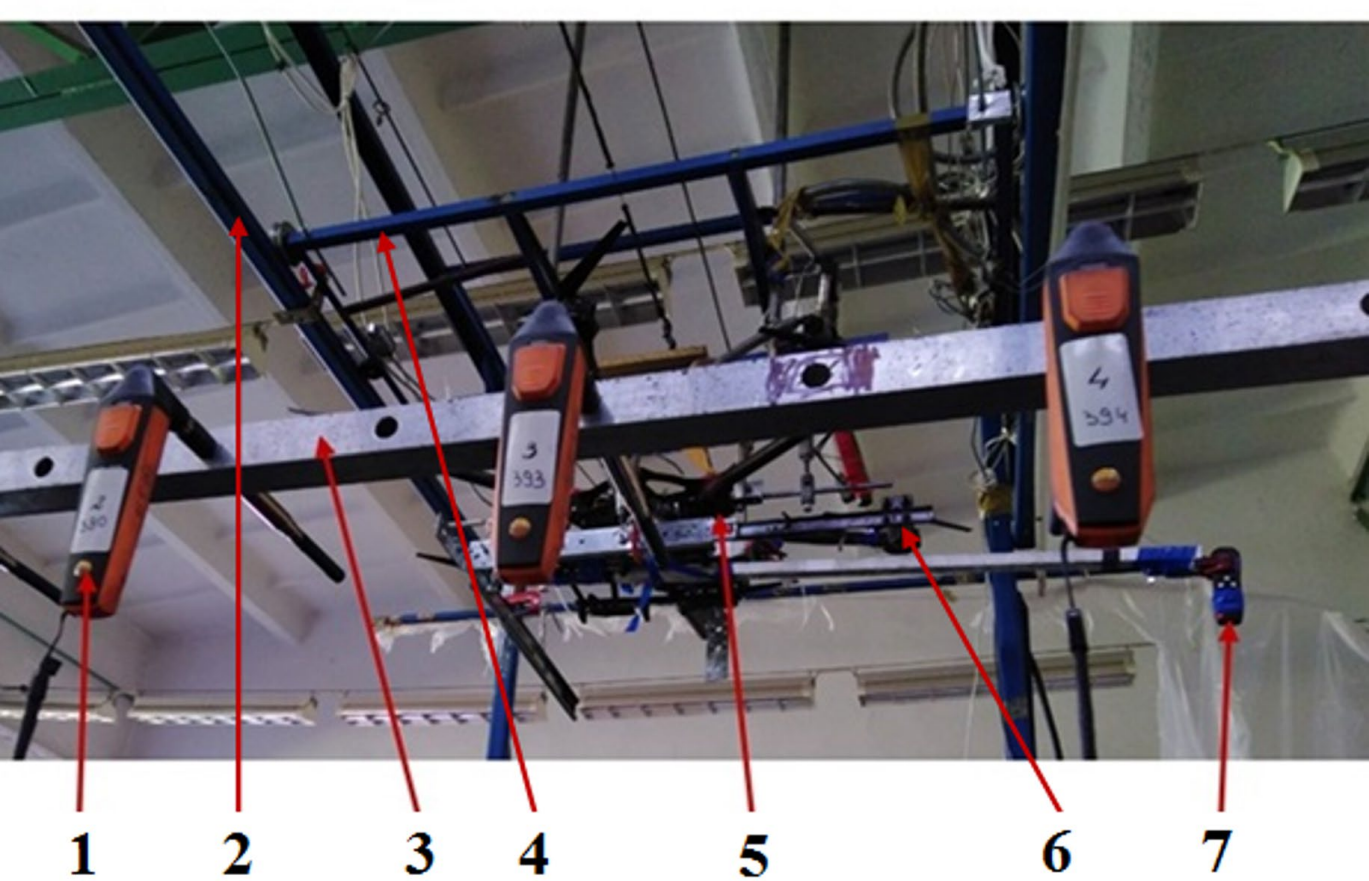
Airflow velocity measurement setup: 1 – anemometers, 2 – track, 3 – beam for mounting anemometers, 4 – trolley, 5 – UAV, 6 – bracket with spray nozzle, 7 – laser pointer for positioning the UAV.

Before spraying the plants, the airflow under the rotors was analyzed. Six Testo 405i anemometers were used to measure airflow velocity. They were mounted on a common aluminum beam set transversely to the UAV’s direction of movement. The anemometers were spaced at intervals of l = 0.20 m. A diagram of the test setup is shown in Fig. 2. The measurement included only the vertical component of the airflow velocity.

The air flow velocity was measured with the drone stationary. An airflow-velocity measurement setup was precisely positioned at the center of the test stand for moving the UAV over plants. The route UAV was divided into five equal sections, each 0.44 m long, in which the unmanned aerial vehicle was positioned stationary for measurements. The UAV’s position was determined using a measuring strip placed under the drone and a laser pointer (7) shining on the strip scale.

The axis of symmetry of the spray nozzle was placed directly above the midpoint between the third and fourth anemometers. In this way, only the part of the air stream in the immediate vicinity of the rotor under which the nozzle was mounted, and the adjacent rotors, was measured. It was assumed that at UAV height not exceeding 1.0 m above the plants, only this part of the air stream affects the shape and capacity of the droplet stream, as well as the uniformity of liquid application to the plants. The beam with mounted anemometers was placed under the nozzle at a distances corresponding to the nozzle height above the plants during spraying, i.e., H = 0.5 m and H = 1.0 m. The tests used a maximum rotor speed of *n* = 6400 rpm.

### Measurement of the transverse distribution of liquid volume in the droplet stream

To evaluate the impact of airflow on liquid concentration in the droplet stream, the transverse distribution of liquid volume beneath the spray nozzle mounted on the UAV was measured. A measurement stand, consisting of three horizontal rods, equipped with liquid collection samplers, was constructed for this purpose. The rods were positioned transversely to the UAV’s direction of motion. The samplers were polyester self-adhesive labels measuring 0.04 m × 0.02 m, affixed to clips mounted on rods at intervals of 0.10 m. The length of the rods was 2.0 m, and the distance between the rods was 0.5 m. Each rod was treated as a separate measuring repeat for the deposited liquid. Using the same spraying parameters, the measurements were repeated twice on this stand, yielding a total of six measurements. The middle of the rods was exactly under the spray nozzle opening. The measurement of liquid distribution was performed at the same pressure in the serving liquid sprayer as during plant spraying. The nozzle height above the rods corresponded to the heights used in the plant spraying tests, with H = 1.0 and H = 0.5 m.

The spray liquid used in the tests of transverse liquid volume distribution and in the tests of liquid application to plants was distilled water mixed with 0.5% additive of nigrosine. After each spraying, the dried samplers with liquid traces were disconnected from the clips. After the test was completed, nigrosine was washed from every single sampler with a fixed volume of 5.0 ml of distilled water. A previously calibrated spectrophotometer was used to determine the dye concentration in the liquid. Based on the dye concentration in the liquid after the sampler was washed and the known dye concentration in the spray liquid, the volume of liquid applied to the sampler’s surface was calculated. For this purpose, formula (1) was used, taking into account that the weight of the dye in relation to the washing liquid was too small to be included in the formula:

1 $$V = \:\frac{{a \bullet \:Vc}}{S}\:,\upmu {\mathrm{l}}$$

 

where: V – volume of liquid applied to the sampler, a – dye concentration in the washing liquid, ppm, Vc – volume of washing liquid, µl, S – dye concentration in the spray liquid, ppm.

### Estimation of the volume of sprayed liquid deposited on plants

**Fig. 3:**
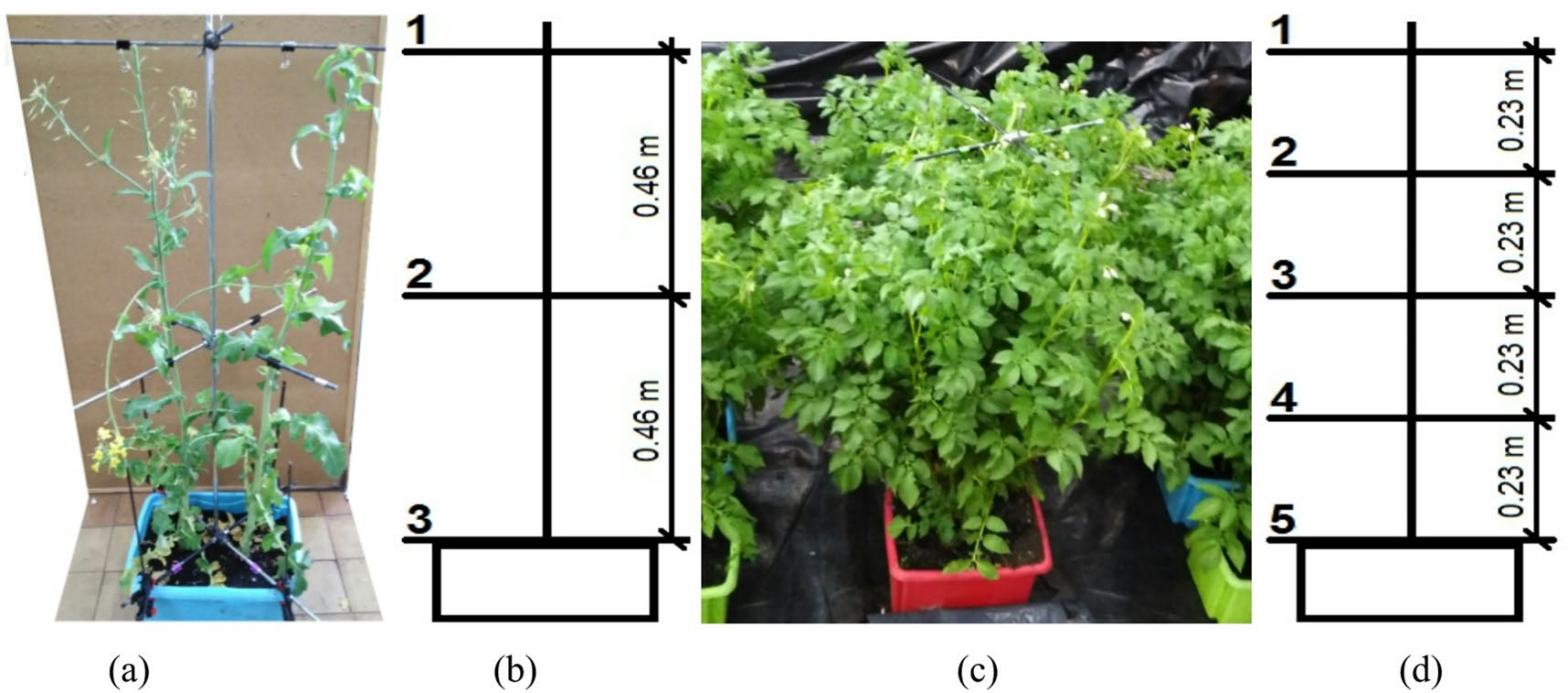
View of the plants and lab stands for liquid application measurement: **a** - rapeseed plants, **b** - dimensions of the measuring stand in rapeseed plants, **c** - potato plant, **d** - dimensions of the measuring stand in potato plant.

The tests used rapeseed plants of the “Lumen” variety and potatoes of the “Tajfun” variety, which are raw materials for food production, and can also serve as direct or waste feedstocks for biofuels and energy production. Investigations of liquid deposition on plants were carried out separately on rapeseed and potato plants. The experiment used 30 rapeseed plants with an average height of 0.97 m and nine potato plants with the same average height. The degree of plant development was preliminarily assessed using the BBCH (Biologische Bundesanstalt Bundessortenamt und Chemische Industrie) scale. The rapeseed was at level 69 (end of flowering – flowering), while the potatoes were at level 60 (beginning of flowering). Plants of both species were grown in the field and then transferred to boxes before the tests. Potato plants were placed individually in each box, whereas rapeseed plants were placed in pairs in each box. Samplers for measuring liquid application on plants were mounted on a lab stand. A vertical stand rod was placed in a single box with each plant species: between two rapeseed plants or in the middle of the potato plant. The sizes of the measuring stands and the assembly method are shown in Fig. 3. Pairs of horizontal rods, crossed at right angles, were attached to the vertical rods at a specific height, corresponding to the measurement level of liquid application. Two clips were placed on each rod at intervals of 0.14 m, symmetrically positioned with respect to the vertical axis of the stand. To these clips, the samplers were glued to capture the sprayed liquid. As the samplers were also used polyester self-adhesive labels, measuring 0.02 × 0.04 m.

The volume of liquid applied to the samplers’ surface was measured simultaneously for all four samplers at each level on the lab stand. Nigrosine from the dried samples was also washed off with 5 ml of distilled water, and the volume of liquid applied to the samplers was determined using formula (1). This value was then normalized to one square centimeter by dividing it by the total surface area of all four samplers. All liquid-applied measurements on plants were repeated five times under the same conditions in each experiment. In Fig. 3, positions (a) and (b) show rapeseed plants and a diagram of a measuring stand with three levels of rods equipped with samples for collecting the liquid applied to the plants. Since the potato plants were more developed in terms of foliage compared to rapeseed, five levels of rods with samples were placed on their stands, as shown in Fig. 3(c) and (d). The boxes containing plants of both species, along with the measuring stands, were placed under the drone so that the axis of the vertical rod of the stand coincided with the center of the spray nozzle.

The boxes containing rapeseed plants were arranged in five rows along the UAV’s path and three rows across it. The box with the measuring stand was positioned exactly in the center between the other boxes. The spacing of the plants in the rows along the direction of movement of the trolley with the UAV was 0.29 m, while the distance between the rows transversely to the direction of travel was 0.19 m.

**Fig. 4:**
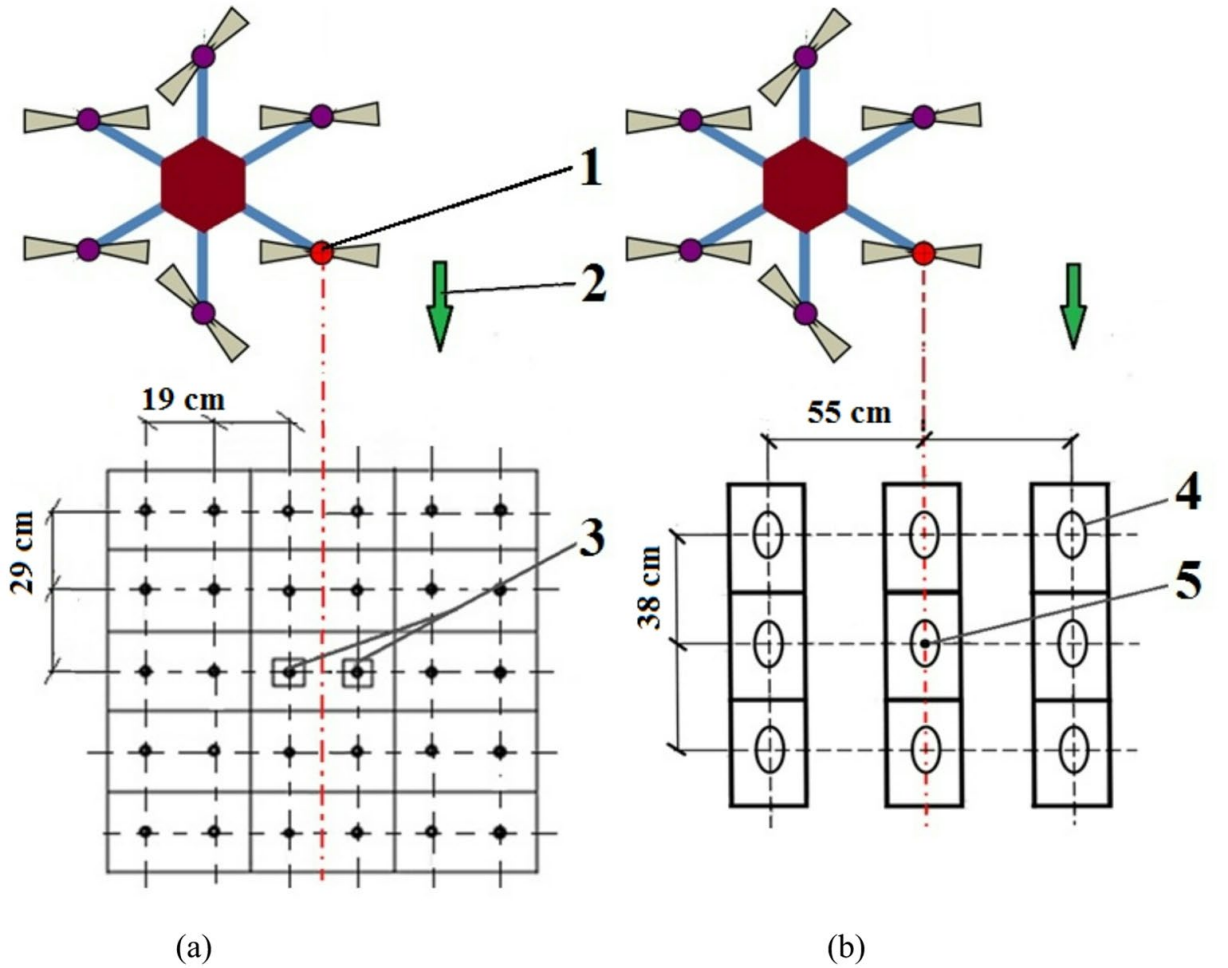
Diagram showing the position of the UAV above the plant boxes: **a** rapeseed, **b** potatoes: 1 – nozzle position on the UAV, 2 – UAV’s movement direction, 3 – rapeseed plants, with a measuring stand (tripod with samplers) placed between them, 4 – potato plant location, 5 – potato plant with a measuring stand with samplers.

The locations of the boxes with rapeseed plants and the measuring stand, relative to the UAV’s trajectory and the spray nozzle, are shown in Fig. 4(a).

The boxes containing potato plants were arranged in three transverse rows and three parallel rows, aligned with the UAV’s direction of travel. The spacing between plants in rows parallel to the UAV’s route was 0.38 m, while the distance between rows transverse was 0.55 m. The positions of the potato plant boxes and the measuring stand placed in the plant boxes, relative to the drone and the spray nozzle, are shown in Fig. 4(b). The plant spacing for both species corresponded approximately to the natural distribution of plants on the plantations from which they were taken.

### Determination of the coverage uniformity coefficient - CU

The uniformity of the liquid plant coverage across all levels was measured using the Coverage Uniformity coefficient (CU). The CU value was calculated on the basis of data from each repetition of the experiment (CU_k_) according to the formula (2):

2 $$\:{\mathrm{C}\mathrm{U}}_{\mathrm{k}}=\frac{1}{{\mathrm{V}}_{\mathrm{s}\mathrm{r}\mathrm{k}}}\sqrt{\frac{\sum\:_{\mathrm{i}=1}^{\mathrm{n}}{({\mathrm{V}}_{\mathrm{i}\mathrm{k}}-{\mathrm{V}}_{\mathrm{s}\mathrm{r}\mathrm{k}})}^{2}}{\mathrm{n}}}$$

where:

CU_k_ – coefficient of coverage uniformity of liquid application at stand levels for the k-th repetition of the measurement; k = 1–5, V_ik_ – the volume of liquid applied to the samplers at the i-th level, at the k-th repetition of the measurement, V_srk_ – average volume of liquid from all samplers at all stand levels for the k-th repetition, n – number of stand levels with samplers.

The CU coefficient value for a given plant at set spraying conditions was calculated after taking into account all CU_k_ results from measurement repetitions.

### Determination of leaf area index (LAI)

To characterize plant foliage and growth, regardless of species or variety, the Leaf Area Index (LAI) was used. After the experiments were ended, all leaves from each plant in the box with a tripod were cut from the space between the first and last levels of the measuring stand. Next, the leaves were scanned, and their total area (cm²) was calculated from the images. The “ground” area under the plants in the LAI calculations was the area defined by the plant arrangement at the test site. The LAI was calculated by dividing the total leaf area by the “ground” area under the plants.

### Statement regarding plants used in research

The authors declare that they did not use endangered plants in their research. The potato and rapeseed species involved in the study are commonly cultivated by farmers in Poland and are not at risk of extinction; therefore, they do not require separate permits for collection and research. These studies were conducted in accordance with relevant institutional, national, and international guidelines and legislation.

### Statistical analysis of the research results

The results obtained in subsequent repetitions of the measurements were subjected to the Shapiro-Wilk test, which showed the normality of the data distribution. The statistical analysis was performed using Statistica version 13.3 by StatSoft. An analysis of variance (ANOVA) was also performed to assess the significance of the effects of UAV movement parameters and the air stream generated by its rotors (propeller rotation) on the volume of liquid at plant levels and on the value of the coverage uniformity (CU) coefficient. Analysis of variance was also used to assess the significance of LAI effects on the CU coefficient.

## Research results and discussion

### Analysis of the air flow velocity

**Fig. 5:**
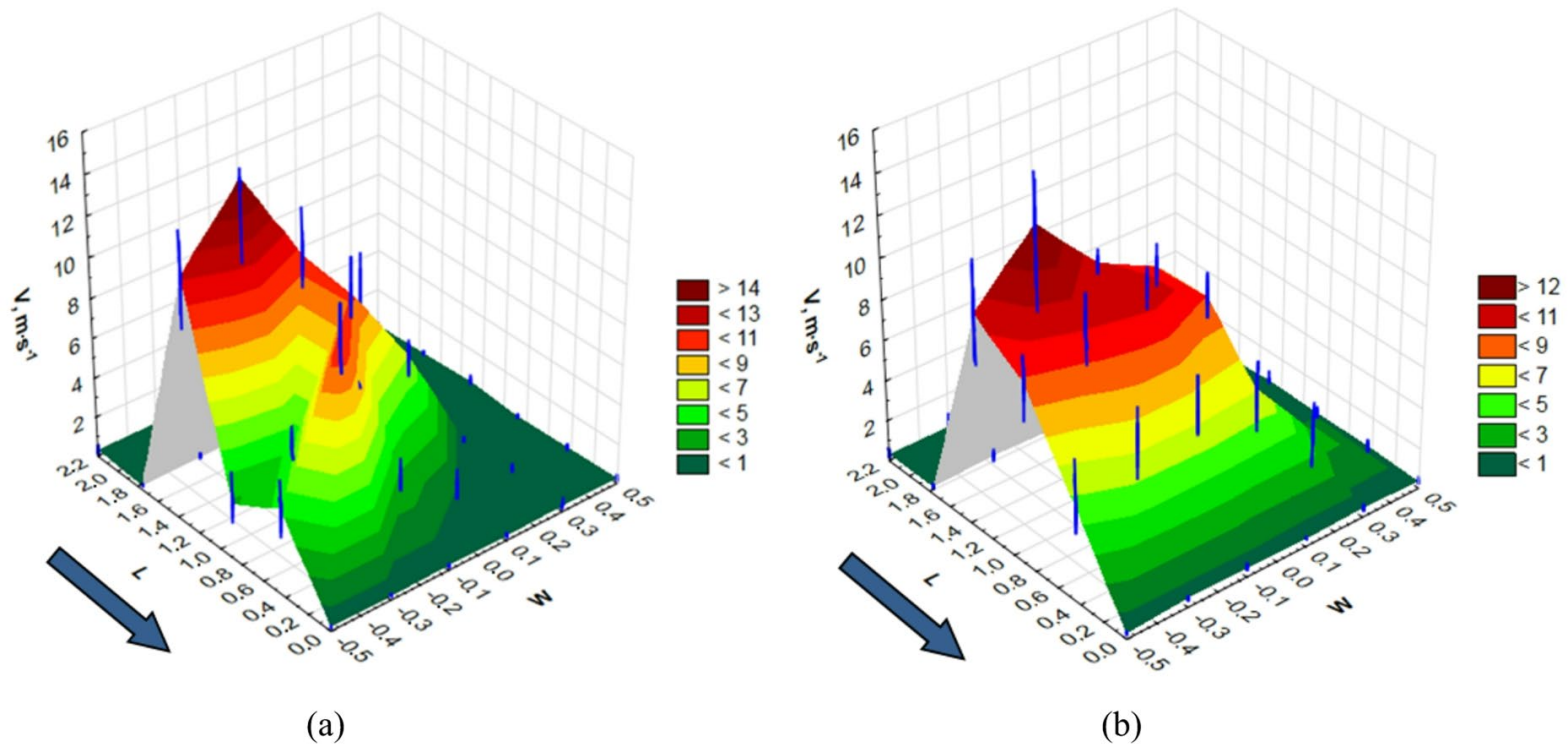
Distribution of air velocity – V, m·s^− 1^ in the measurement space of the test stand at a drone propeller speed of *n* = 6400 rpm: **a** height above plants – H = 0.5 m, **b** height above plants – H = 1.0 m.

Based on the results from the airflow-velocity measurement setup (Fig. 2), a three-dimensional plot was created to depict the distribution of average air velocities. The measurements were taken only on a part of the UAV, in the vicinity of the rotor arm, under which a spray nozzle was mounted. These graphs are shown in Fig. 5.

The graphs also show the UAV’s direction of motion on the test stand, indicated by blue arrows. The average air velocity values are presented topographically as levels. The measurement points are marked with thin blue vertical lines. The length of these lines indicates the spread of the results.

The longitudinal direction of the UAV’s movement is marked with the letter L, and the transverse section with the letter W. Values above the “0” point on the W axis, along the L axis, correspond to the air velocities on the line of movement of the spray nozzle mounted under the arm. The graph also shows an increase in airflow behind the nozzle due to the rotation of the second UAV rotor. Areas near the rotors, where the airflow velocity is highest, are visible, as well as a sharp decrease in velocity with increasing distance from the rotors. A comparison of the air velocity graphs for the lower UAV height (H = 0.5 m) and the higher height (H = 1.0 m) shows that the higher height not only yields a lower air velocity at the plant level but also reduces the velocity differences within the stream. Analysis of the graphs also highlights the important role of the nozzle’s position relative to the UAV’s rotors.

### Transverse distribution of liquid volume in the droplet stream

**Fig. 6:**
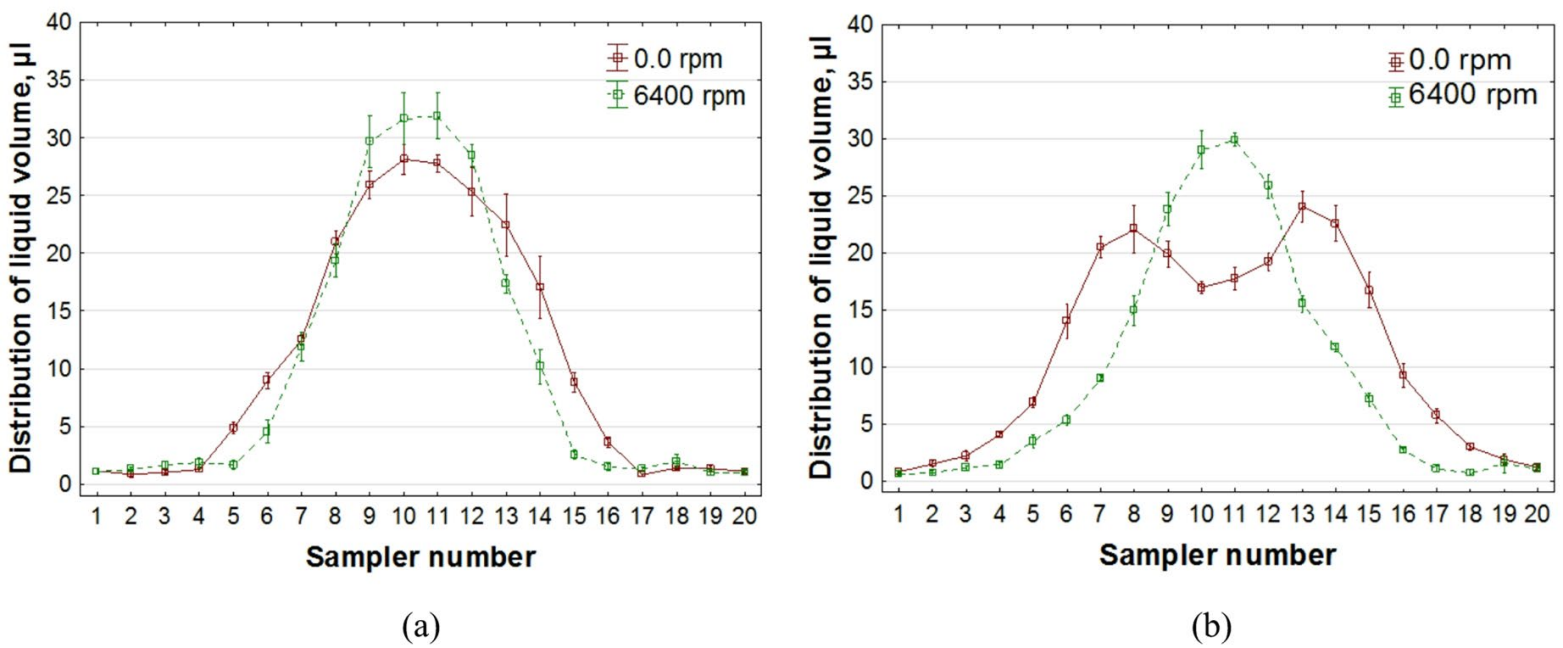
Graph showing changes in the distribution of liquid volume under a nozzle mounted on the UAV as a result of the impact of the airflow: **a** nozzle height above the samplers – H = 0.5 m, **b** nozzle height above the samplers – H = 1.0 m.

Figure 6 shows graphs illustrating how the liquid volume distribution under the nozzle changes due to the impact of the air stream. The burgundy color indicates results obtained with the rotors stationary and no airflow, whereas the green color shows liquid deposition on the samplers when airflow from the UAV rotors at maximum propeller speed (*n* = 6400 rpm) is present.

The graphs primarily illustrate the change in the liquid volume traverse distribution under the nozzle, as a function of the nozzle height above the plant surface in the absence of airflow and with airflow. Increasing the nozzle height from 0.5 to 1.0 m above the measurement surface not only broadens the distribution width but also significantly reduces the volume of deposited liquid in the central part of the stream. The flat fan spray nozzle used is designed for spraying crops from the level of a field sprayer boom, with a standard nozzle height above the plant surface of H = 0.5 m. At this height, the largest amount of liquid is deposited in the central part of the stream (Fig. 6(a)). However, at a height of H = 1.0 m above the plant surface, a so-called “saddle” formed in the central part of the distribution, indicating a reduced volume of the applied liquid (Fig. 6(b)).

The airflow generated by the UAV’s rotors reduced the spray angle and narrowed the width of the liquid deposited on the probes by approximately 20%. In addition, the value of liquid volume in the central part of the stream increased (Figs. 6(a) and (b)), which is the result of the acceleration of the vertical component of the droplet fall velocity under the influence of air momentum. The largest increase in liquid volume was observed in the central part of the chart, at a height of H = 1.0 m. The acceleration of the liquid’s fall at this height eliminated the “saddle” and significantly increased the volume of liquid applied in this region of the droplet stream. This reduction in the liquid spray angle from the nozzle, under the influence of UAV-generated airflow, as well as changes in the stream’s volume, were also observed in studies^35^.

Research has shown that increasing the volume of liquid in the central region of its transverse distribution, when spraying plants with a single nozzle, enables the spray to be concentrated on selected plants. To perform precise spraying on individual plants, the nozzle height should be lowered. The air stream from the UAV rotors, which narrows the transverse distribution, supports the precision of the treatment. Increasing the flight altitude widens the transverse distribution, allowing preventive spraying of plants located to the side of the sprayed plants and also within the remaining part of the width distribution.

### Liquid application volume at the plant levels

**Fig. 7:**
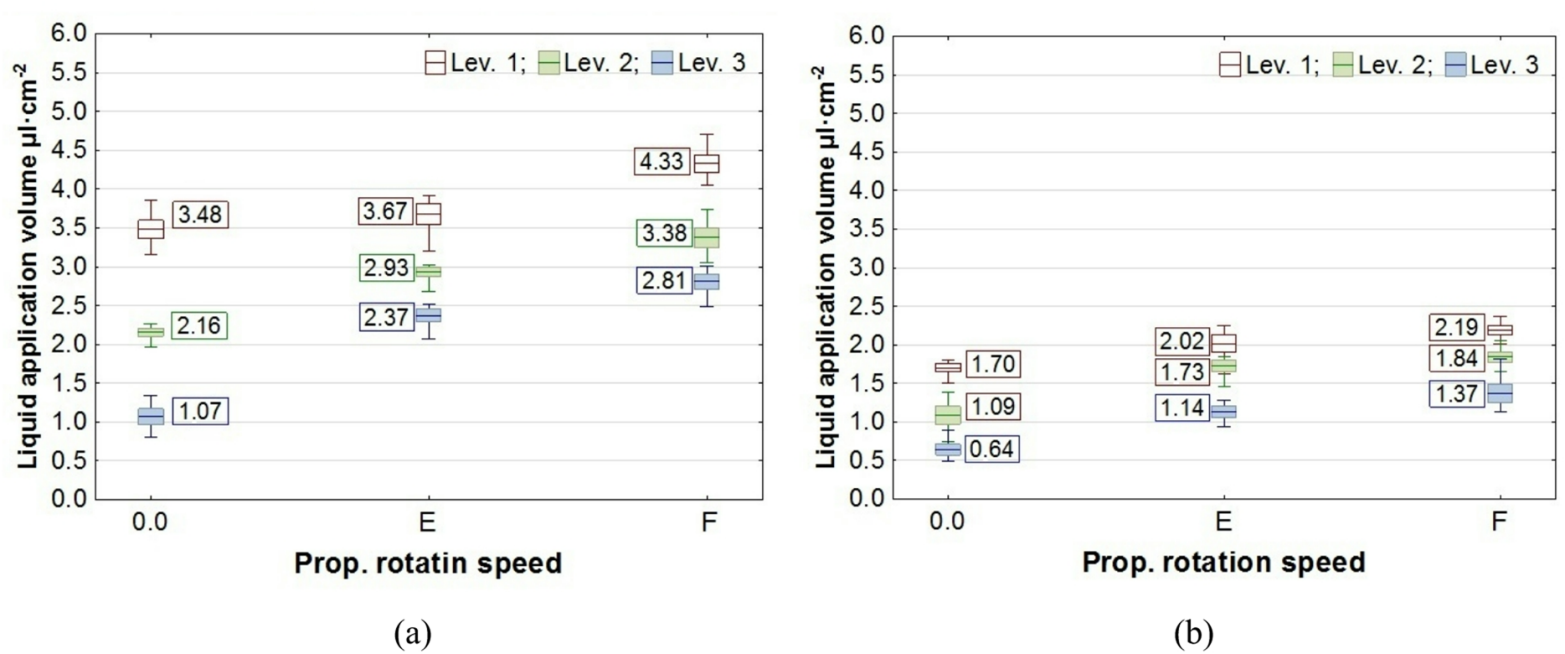
The effect of propellers’ rotation speed on the volume of liquid applied to rapeseed plant levels at the UAV’s altitude of H = 0.5 m: **a** v = 0.54 m·s^− 1^, **b** v = 1.00 m·s^− 1^.

**Fig. 8:**
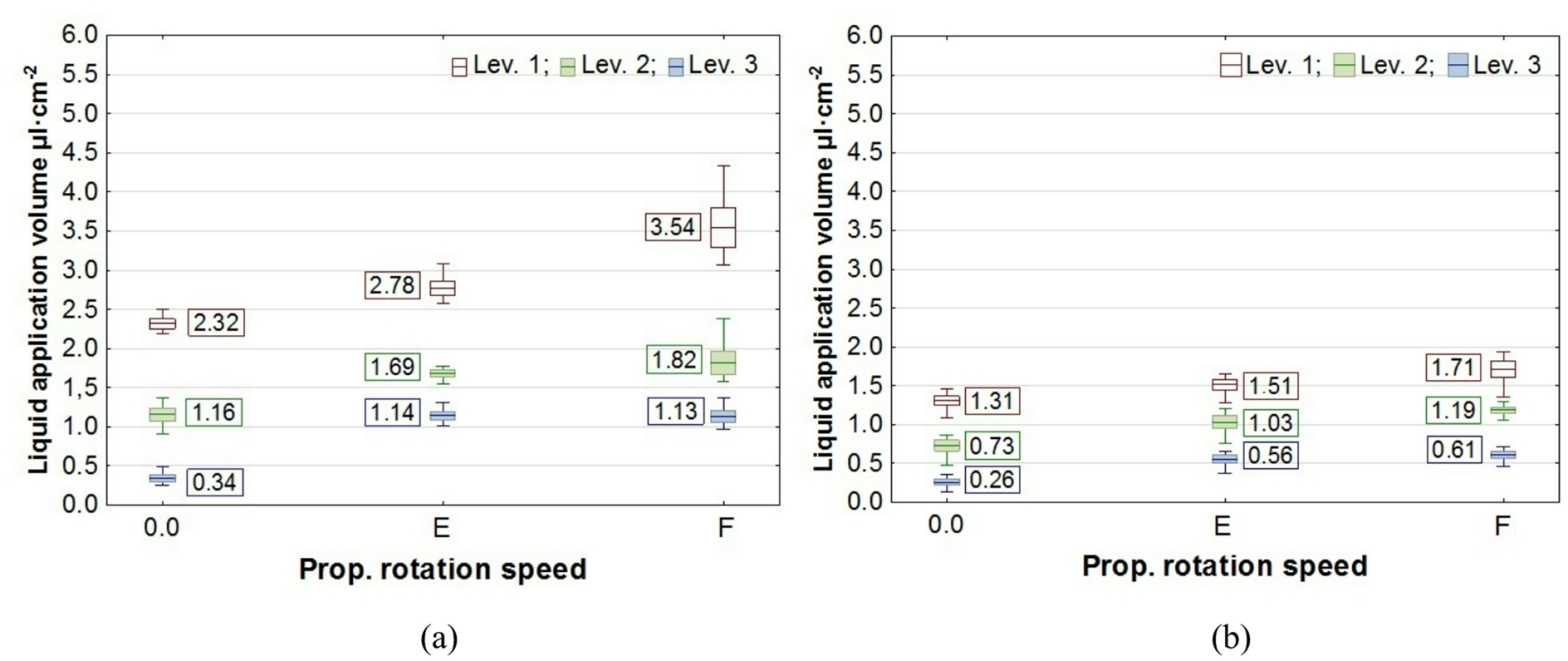
The effect of propellers’ rotation speed on the volume of liquid applied to rapeseed plant levels at the UAV’s altitude of H = 1.0 m: **a** v = 0.54 m·s^− 1^, **b** v = 1.00 m·s^− 1^.

**Fig. 9:**
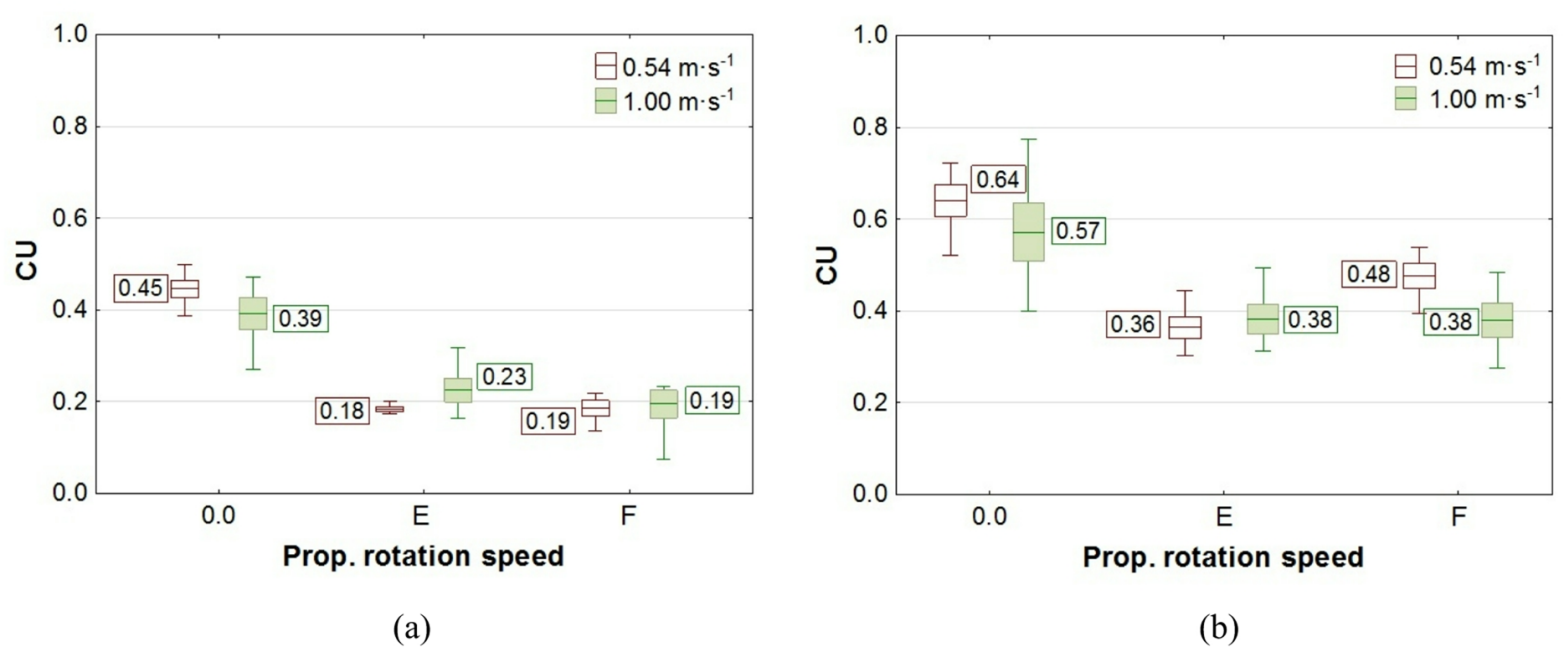
Effect of propellers’ rotation speed on the coverage uniformity coefficient - CU in rapeseed plants at flight height: **a** H = 0.5 m, **b** H = 1.0 m.

**Fig. 10:**
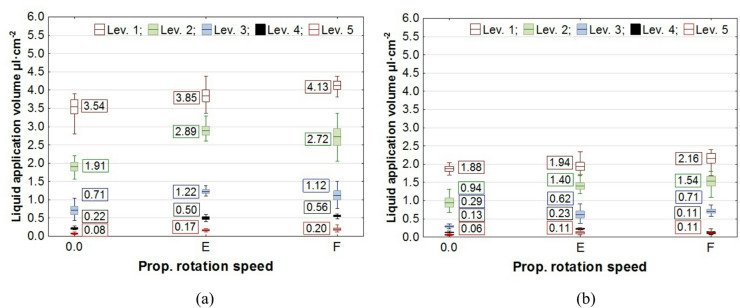
The effect of propellers’ rotation speed on the volume of liquid applied to potato plant levels at the UAV’s altitude of H = 0.5 m: **a** v = 0.54 m·s^− 1^, **b** v = 1.00 m·s^− 1^.

**Fig. 11:**
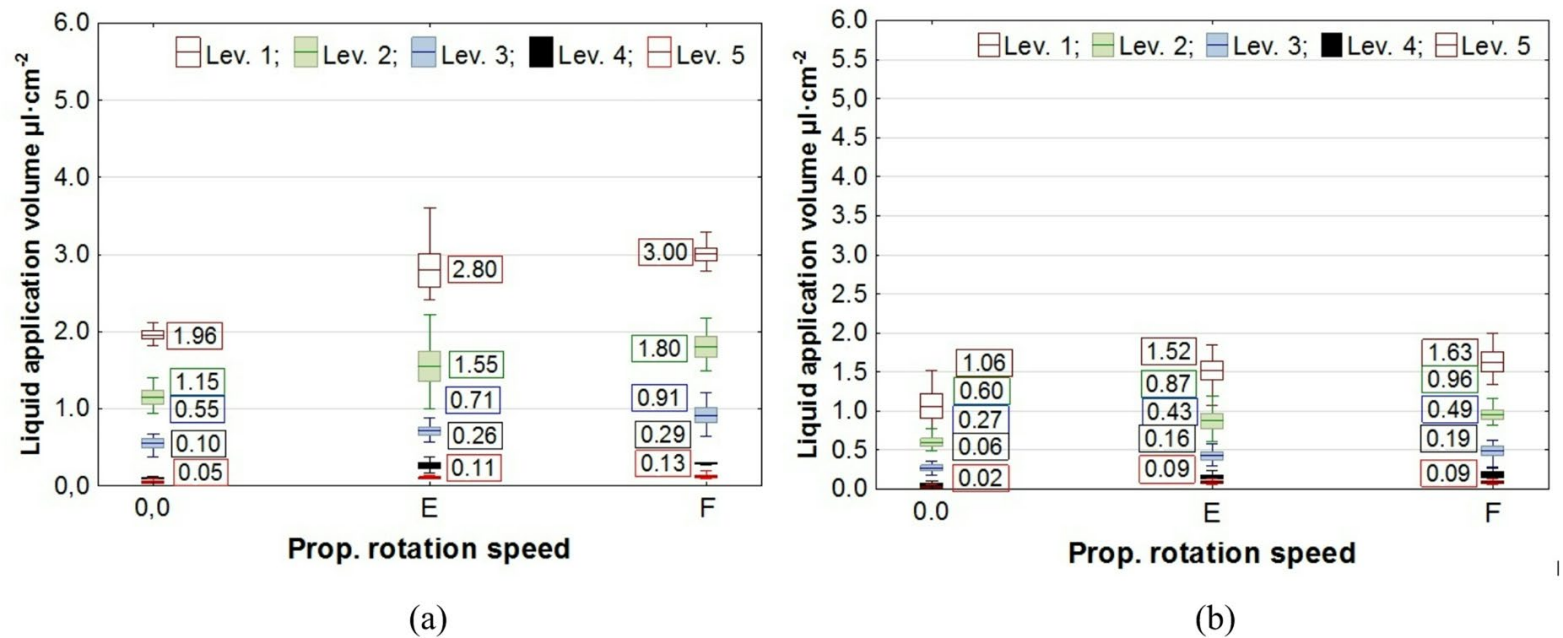
The effect of propellers’ rotation speed on the volume of liquid applied to potato plant levels at the UAV’s altitude of H = 1.0 m: **a** v = 0.54 m·s^− 1^, **b** v = 1.00 m·s^− 1^.

**Fig. 12:**
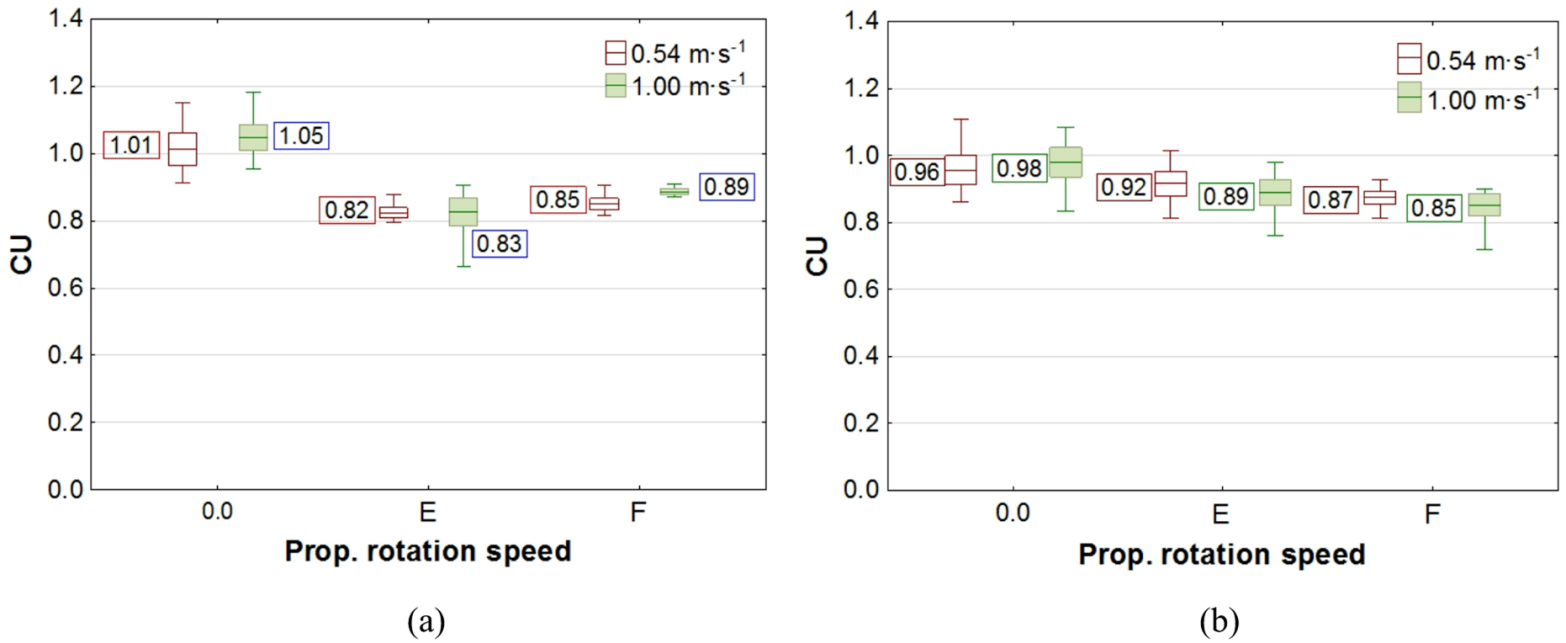
Effect of propellers’ rotation speed on the coverage uniformity coefficient - CU in potato plants at flight height: **a** H = 0.5 m, **b** H = 1.0 m.

The results of the study on liquid application volume across all plant levels, along with the values of the coverage uniformity coefficient (CU), are presented in box plots. In the charts, the center line indicates the mean value, the upper and lower levels of the box correspond to ± standard error, while the whiskers represent the maximum and minimum values obtained in repeated measurements. On the chart “Propeller Rotation Speed” axis, the value “0.0” corresponds to no rotation of the UAV’s rotors, the value “E"─ “Empty”, corresponds to the rotation of the propellers for the UAV with an empty spray tank, and the value “F"─ “Full”, corresponds to the rotation of the rotors with a full tank. The volume of liquid deposited on all levels of rapeseed plants, depending on the UAV’s propeller rotation speed, is shown in Figs. 7 and 8, while the corresponding coverage uniformity coefficient - CU values are shown in Fig. 9. Similar results for potato plants are presented in Figs. 10 and 11, and the CU values in Fig. 12.

The liquid coverage uniformity coefficient (CU) was calculated for each measurement repetition and for each individual case, both with and without airflow. Therefore, the values shown in the box plots for CU also include the calculated mean, standard error, and minimum and maximum values, as in the liquid application plots.

Analysis of how airflow (propellers’ rotation speed) affects liquid application showed that it enhances liquid penetration into plants, increasing the amount of liquid on levels 2 and 3 for rapeseed plants and on levels 2 to 5 for potato plants.

A notable phenomenon was an increase in the amount of liquid applied at the topmost level of the samplers, which corresponds to the height of the plant tops. This effect was caused by the design of the measuring stands and the positioning of plants on stands relative to the nozzle (Figs. 3 and 4). As shown by analysis of the liquid distribution under the nozzle, the air stream increased the volume of liquid in the central part of the stream, where the samplers on the first and subsequent levels were positioned at a distance of 0.14 m from the stand’s axis.

Consequently, there was not only an increase in liquid volume at the upper level, but also an improvement in application coverage of the lower parts of the plants. This raises the question of whether the increase in dose to the lower parts results solely from the higher concentration of the liquid stream in its center or from an overall improvement in application uniformity along the entire height of the plants. The results of the coverage uniformity coefficient (CU) for both species showed that the value decreased at heights H = 0.5 m and H = 1.0 m for both UAV speeds when the rotors were spinning, regardless of UAV load, compared with the case when the rotors were not spinning. The decrease in CU value in the presence of an airflow indicates an improvement in the uniformity of liquid application to plants caused by the airflow generated by the rotors. Due to the higher density of potato foliage, the variation in the applied dose to individual levels was much greater than in the case of rapeseed, resulting in CU values for potatoes exceeding even 1.0. A comparison of the two UAV moving speeds showed that the amount of liquid applied was directly proportional to the UAV speed, while no significant effect from reducing the speed on the CU coefficient improvement was observed in any of the species. However lowering the spraying altitude from H = 1.0 m to H = 0.5 m significantly improved the uniformity of liquid application in rapeseed plants, as indicated by a decrease in the CU value.

### Significance of factors affecting spray liquid application

To assess the significance of the airflow generated by unmanned aerial vehicle rotors on the quality of liquid application to plants, a statistical analysis was performed, and p-values for the coefficient of significance were calculated. The tests examined how the airflow produced by rotating rotors (propeller speeds of 5000 and 6400 rpm) affects the volume of liquid applied to plant levels and the coverage uniformity coefficient (CU), compared with spraying plants without an air stream (propeller speed of 0.0 rpm). Tests were also conducted to assess the significance of the difference in airflow between an empty tank (propeller rotation speed of 5000 rpm) and a full tank (propeller rotation speed of 6400 rpm). Significance tests were performed for all UAV heights and speeds. The application of liquid across all plant levels was accounted for in the calculation of the p-value. The results, shown in Tables 1 and 2 (column 3) and Table 3 (columns 3 and 4), indicate the significance of airflow (results for propeller rotation speeds of 5000 and 6400 rpm were taken together) compared with spraying without airflow (rotor speed of 0 rpm) on the results of liquid application to plants. The values in Tables 1 and 2, column 4, present the p-values for airflow velocity, comparing cases with the UAV’s tank empty (5000 rpm) and full (6400 rpm). The analysis of the significance of airflow’s impact on the liquid coverage uniformity coefficient (CU) for both rapeseed and potato plants is shown in Fig. 3. The p-values below the significance level of α = 0.05 are highlighted in bold as significant.

**Table 1:** The p-values for the liquid volume deposited in the rapeseed plant levels.

Height of the UAV above the plants H, m	UAV speed over plants v, m·s^− 1^	The *p*-values when comparing the volume of liquid deposited at plant levels during spraying with and without propeller rotations			The *p*-values for comparisons of the liquid volume deposited at plant levels during spraying with an empty and a full tank		
		3			4		
1	2	Lev. 1	Lev. 2	Lev. 3	Lev. 1	Lev. 2	Lev. 3
0.50	0.54	**0.0009**	**0.0000**	**0.0000**	**0.0060**	**0.0130**	**0.0093**
	1.00	**0.0035**	**0.0001**	**0.0004**	0.2136	0.3018	0.1411
1.00	0.54	**0.0003**	**0.0002**	**0.0000**	0.1559	0.1237	0.4045
	1.00	**0.0127**	**0.0013**	**0.0003**	**0.0229**	0.4200	0.8885

**Table 2:** The p-values for the liquid volume deposited in the potato plant levels.

Height of the drone above the plants H, m	Drone speed over plants v, m·s^− 1^	The *p*-values when comparing the volume of liquid deposited at plant levels during spraying with and without propeller rotations					The *p*-values for comparisons of the liquid volume deposited at plant levels during spraying with an empty and a full tank				
		3					4				
1	2	Lev. 1	Lev. 2	Lev. 3	Lev.4	Lev. 5	Lev. 1	Lev. 2	Lev. 3	Lev.4	Lev. 5
0.50	0.54	0.0755	**0.0028**	**0.0085**	**0.0000**	**0.0112**	0.199	0.5428	0.4613	0.217	0.5045
	1.00	0.1796	**0.0104**	**0.0014**	**0.0028**	0.1571	0.2303	0.4022	0.4385	**0.0073**	0.9422
1.00	0.54	**0.0004**	**0.0265**	**0.0128**	**0.0001**	**0.0084**	0.3999	0.3329	0.1032	0.4841	0.3359
	1.00	**0.0301**	**0.0129**	**0.0220**	**0.0168**	**0.0029**	0.557	0.4919	0.4293	0.4699	0.8927

**Table 3:** The p-values for the coverage uniformity coefficient (CU) in the levels of rapeseed and potato plants.

Height of the drone above the plants H, m	Drone speed over plants, v, m·s^− 1^	The *p* value, when comparing the coverage uniformity coefficient (CU) during spraying without and with propellers rotations	
		Rapeseed plants	Potato plants
1	2	3	4
0,50	0,54	**0.0000**	**0.0016**
	1,00	**0.0013**	**0.0015**
1,00	0,54	**0.0000**	0.2861
	1,00	**0.0000**	0.1000

The statistical analysis of the research test results showed that the airflow generated by the UAV’s rotors, compared with the absence of airflow, significantly affected the amount of liquid deposited on rapeseed and potato plants. The calculated p-values were all below the significance level α = 0.05 for the rapeseed plants. For potato plants, three cases at a height of H = 0.5 m had p-values exceeding the significance level α = 0.05. These results might be considered outliers, possibly due to insufficient measurement replicates. Comparing the significance of airflow resulting from propeller rotation speeds for UAV with an empty and full tank revealed a notable effect of airflow on liquid deposition volume, observed only on rapeseed plants at UAV flight height H = 0.5 m and a speed of v = 0.54 m·s^− 1^. At height H = 1.0 m, the p-value of 0.0229 was less than α = 0.05, but this was only observed during the first level of samplers and at a speed of v = 0.54 m·s^− 1^, which may also indicate a random occurrence.

The analysis of airflow’s impact on the liquid coverage uniformity coefficient (CU) showed a significant effect only on sprayed rapeseed plants at both studied heights and speeds of UAV movement, but only when comparing liquid depositions with rotors rotating to results without rotor rotation. For potato plants, a similar significance of airflow’s impact on CU was observed only at H = 0.5 m for both drone speeds.

The analysis results indicate that when using multi-rotor UAVs for crop spraying, the generated airflow can substantially affect the uniformity of liquid distribution to crops. This effect is especially pronounced when spraying at low UAV flight altitudes and at slow speeds.

### Leaf area index

LAI values were calculated based on the area of leaves cut from plants and scanned, as well as the calculated area of the “ground” beneath the plants. For rapeseed plants, the “ground” area was the area of the box (1102 cm²) on which the measuring tripod was placed. For potato plants, the field area per plant was 2090 cm², determined by plant spacing. The total leaf area and LAI results for both plant species are shown in Table 4.

**Table 4:** LAI values for rapeseed and potato plants.

Plants	Total leaf area, cm^2^	Leaf area index - LAI
Oilseed	966.75	0.877
Potato	13110.75	6.273

In the case of rapeseed, a lower LAI was associated with lower leaf density and greater interleaf spacing than in potato plants. This significantly influenced the effect of airflow from the unmanned aerial vehicle (UAV) on increasing the volume of liquid deposited on the lower levels of the plants, thereby improving CU compared to the application of liquid there in the absence of airflow. In this case, differences in rotor speed, caused by tank filling, also significantly affected the amount of liquid applied to the low levels of the rapeseed canopy (Table 1). The high LAI value for potato plants resulted in very dense foliage, which hindered penetration of the liquid into the lower layers. The high LAI value reduced the impact of the air stream on the volume of liquid application for all spraying parameters and on its significance for the CU coefficient.

To identify the most significant factors affecting the coverage uniformity coefficient (CU), an ANOVA of main effects was performed on the obtained CU data using Statistica. Only results from spraying tests with UAV propeller speeds, corresponding to an empty and a full tank of liquid were included in this analysis. The analysis showed only the significance of the impact of the LAI coefficient of plants and the spraying height H (p ˂ 0.05).

The analysis did not show any effect of propeller rotation speed and UAV movement speed (v).

A second, similar analysis was conducted on the CU results, but only for those obtained without propeller rotations. This analysis showed that, in the absence of airflow beneath the drone, only the LAI coefficient significantly influenced the CU values.

Since previous analyses of the significance of other factors’ impact on the CU value for individual cases showed that the UAV’s speed of movement (v) and rotor speed, which vary with an empty or full tank, may also be influential, a multifactorial analysis of variance was conducted to examine the significance of all parameters of spraying plants from the unmanned aerial vehicle, at rotor speeds with an empty and full tank, and LAI. The analysis results are shown in Table 5.

**Table 5:** The p-values indicating the significance of the studied factors and their correlations to the coverage uniformity coefficient, CU.

Factors		*p*-value
1	H	**0.000000**
2	LAI	**0.000000**
3	Prop. rot. speed	**0.001811**
4	v	**0.034744**
5	H & v	**0.000045**
6	LAI & H	**0.000000**
7	LAI & v	**0.009893**
8	LAI & H & v	**0.040978**
9	Prop. rot. speed & H	**0.008360**
10	Prop. rot. speed &LAI	0.070083
11	Prop. rot. speed & v	0.135351
12	Prop. rot. speed & H & v	**0.000836**
13	Prop. rot. speed & LAI & H	**0.000003**
14	Prop. rot. speed & LAI & v	**0.000111**
15	Prop. rot. speed & LAI & H & v	**0.047099**

The multivariate analysis showed an additional possibility of a significant impact on CU values of propeller rotation and UAV movement speed - v. It also demonstrated the potential significant impact of interactions: height H and v, LAI and H, LAI and H and v, and propeller rotation in combination with H, with H and v, with LAI and H, with LAI and v as well as the importance of propeller rotation interaction with all other factors. In these studies, no significant interaction between propeller rotation and LAI, or between propeller rotation and UAV travel speed, was observed for CU values (*p* > 0.05). For a similar multifactoral significance analysis performed in the absence of propeller rotation, the multivariate analysis showed significant effects only for the LAI coefficient, spraying height (H), and the interaction between LAI and H.

### Experimental limitations

The drift of spray liquid sprayed by aircraft was a problem in typical manned agricultural aviation. This problem remains relevant when spraying with unmanned aerial vehicles (UAVs), particularly when treatments are conducted at high altitudes. The possibility of uncontrolled contamination of the natural environment with drifted chemicals led the European Parliament and the Council to include restrictions on aerial spraying in the Directive on the sustainable use of pesticides - Directive 2009/128/EC^90^. Therefore, research on the assessment of the quality of liquid application to plants using UAVs, assuming that in the future these will be flying robots performing precise treatments, i.e., only on selected plants or groups of plants, from a low height, typical for the boom of a field sprayer. It was recognized that only in that manner could aerial spraying support the sustainable use of pesticides.

Undertaking such research without prior knowledge entailed taking and accepting experimental limitations in the parameters used for spraying and in the plants on which the research was conducted. For this study, the research was conducted at heights and UAV flight speeds that are not commonly used in studies and practice. Therefore, extreme parameter values were selected to maximize the detectability of the effect and to establish the boundary conditions for the phenomenon under study. The omission of intermediate values was intentional, given the exploratory nature of the study. This approach resulted in two levels of altitude and speed parameters: only the lower and upper levels. This approach also influenced the selection of plant species for testing. The selected rapeseed and potato plants were at similar developmental stages and heights but differed significantly in LAI and morphology. It was also observed that rapeseed plants, in addition to having a lower LAI than potato plants, were more delicate and more prone to bending under airflow, whereas potato plants had stiffer, thicker stems and leaves and did not bend. Therefore, it can be inferred that, in addition to LAI, plant structure also influenced how liquid droplets reach the lower parts of the plants.

The airflow and its impact on the distribution of liquid volume under a nozzle were studied following spraying tests, in which liquid was applied at various levels of the plant. This was done to explain the observed increase in liquid coverage across all plant levels when spraying with a UAV with rotating propellers, compared with spraying without propeller rotation. To determine the boundary conditions for this phenomenon, the distribution of liquid volume beneath the nozzle was measured at two conditions: maximum propeller speed and no propeller rotation, at two heights. At two heights, the distribution of air velocity was also pointed out under the nozzle. 

### Suggestions for future research

Given the limited scope of these experiments, further research is necessary to better understand the factors that influence the effectiveness of using unmanned aerial vehicles (UAVs) as autonomous flying robots at low altitudes for precision plant care. Future studies should consider:

- Increasing the diversity of plant species included, considering indirect LAI values and differences in their morphology and structure, especially within the boundary parameters range used in this study;
- Analyzing the use of different types and sizes of spray nozzles, particularly air injection nozzles, and identifying the most suitable options for treating individual plants at low UAV flight altitudes;
- Conducting tests to assess the accuracy of liquid application by placing numerous samples at various distances from the spray nozzle’s axis, across the drone’s movement, to check coverage of at least the width of the liquid deposition strip. Additionally, consider a wider range of spray parameters by including not only extreme values but also intermediate values.

### Summary

the airflow generated by the drone’s rotors significantly affected the volume and precision of liquid application to plants.

The effect of the air stream was:

- an increase of liquid volume in the central part of the droplet stream under the nozzle,
- narrowing of the width of the transverse distribution of liquid at the level of the sprayed plants,
- increase in the amount of liquid deposited in the lower parts of plants,
- improvement in the liquid coverage uniformity coefficient on plants.

Research shows that the distribution of liquid volume in the droplet stream beneath the nozzle varies not only with the air flow induced by the propellers’ rotation but also with the drone’s altitude above the plants. These factors can affect the quality of plant spraying if they are not accounted for. Research has demonstrated the usefulness of the LAI (Leaf Area Index) as an independent, physical-mathematical parameter that characterizes plant foliage and growth. These advantages of LAI make it valuable for assessing the effectiveness of spraying and for planning precision spraying of plants using UAVs. Both plant species used in the study were of the same height, but rapeseed plants, with a lower LAI than potato plants, allowed for a greater volume of liquid to be deposited at lower levels. This resulted in improved uniformity of liquid application to these plants and a lower coverage uniformity coefficient (CU) value.

The (ANOVA) analysis of main effects showed that the (CU) liquid coverage uniformity coefficient from UAVs to plants is mainly affected by plant foliage density, measured by LAI and UAV altitude. That means that airflow from the rotating propellers, as the UAV descends, causes greater deflection of the leaves and shoots, creating gaps through which droplets are blown into the interior of the plant rhizomes.

Additionally, other morphological and mechanical characteristics of plants could be important, such as leaf shape and structure, their arrangement along the stem, the shape, size, and stiffness of stems, and potentially the anatomical structure of stems.

## Conclusions

Based on the research conducted, the following conclusions can be drawn about the factors affecting the accuracy and quality of liquid application on plants sprayed with a single nozzle mounted on a multi-rotor drone flying at low altitudes:

1. Effect of air stream – The airflow from the UAV’s rotors narrowed the droplet stream produced by the single nozzle by about 20% and increased the liquid volume in the center of the droplet stream, especially at a UAV height of H = 1.0 m. It also enhanced the application of liquid to the lower rhizomes of plants.
2. Importance of UAV flight altitude – The UAV’s altitude is a critical factor influencing the amount of liquid applied to the leaves and the consistency of plant coverage. Lower altitudes (H = 0.5 m) improve the uniformity of liquid application to plants and enable deeper penetration into dense foliage. Additionally, at very low UAV altitudes and speeds, propeller rotation speed, depending on the tank’s liquid level, may influence the amount of liquid applied to plants. The low height of an unmanned aerial vehicle, comparable to the average height of a ground-based field sprayer boom, will enable, in the future, precise spraying of individual plants, particularly when UAVs are used as robots in cultivated fields.
3. Significance of the LAI – The Leaf Area Index (LAI) is a useful tool for predicting and evaluating the quality of plant spraying with a UAV. It accounts for leaf density across plant types, helping to predict the appropriate distribution of liquid. Observations indicate that high LAI values can significantly impede liquid penetration into the lower parts of plants and impair the uniformity of liquid application.
4. Nozzle selection – Selecting the appropriate nozzle type and size requires research that accounts for both the impact of the air stream and the UAV’s flight altitude on the distribution of liquid volume under a nozzle and on the spray precision on plant canopies.

## Data Availability

https://doi.org/10.5281/zenodo.17221483
